# Real-Time Learning and Recognition of Assembly Activities Based on Virtual Reality Demonstration

**DOI:** 10.3390/s21186201

**Published:** 2021-09-16

**Authors:** Ning Zhang, Tao Qi, Yongjia Zhao

**Affiliations:** 1 State Key Laboratory of Virtual Reality Technology and Systems, School of Automation Science and Electrical Engineering, Beihang University, Beijing 100191, China; zy1703239@buaa.edu.cn (N.Z.); qitao@buaa.edu.cn (T.Q.); 2 Jiangxi Research Institute, Beihang University, Nanchang 330096, China

**Keywords:** virtual reality, activity recognition, learn from demonstration

## Abstract

Teaching robots to learn through human demonstrations is a natural and direct method, and virtual reality technology is an effective way to achieve fast and realistic demonstrations. In this paper, we construct a virtual reality demonstration system that uses virtual reality equipment for assembly activities demonstration, and using the motion data of the virtual demonstration system, the human demonstration is deduced into an activity sequence that can be performed by the robot. Through experimentation, the virtual reality demonstration system in this paper can achieve a 95% correct rate of activity recognition. We also created a simulated ur5 robotic arm grasping system to reproduce the inferred activity sequence.

## 1. Introduction

Due to the complex and boring industrial environment of modern industry, it is of great practical significance to complete assembly tasks by robots instead of humans. Traditional robots require users to have programming skills, which gives robots capabilities beyond that of the general public. Currently, these robotic arms can only handle simple tasks. The operation skills of the agent are mostly generated by manual hard programming, which requires a lot of labour and debugging. When the production line is optimised and adjusted, a lot of manual operation skills are also required.

Today, robotics researchers are studying a new generation of robots that can learn from human demonstrations without programming [1,2]. In other words, these new types of robots can use sensors to perceive human movements and imitate human movements [3], which can greatly accelerate the deployment efficiency of robots in new environments and new tasks. Learning the human-like operating skills required to complete complex tasks through the demonstration of “observing” human experts and realising the migration of human skills to robot agents are hot topics in the field of intelligent robots and have important theoretical significance and application value. The core of demonstration learning is to let the agent understand the behavioural intention of the demonstrator in order to achieve a specific task goal and have the ability to reproduce the task. In recent years, robot imitation learning [4] represented by behavioural cloning [5], inverse reinforcement learning [6] and generative adversarial network [7] methods has made certain progress, laying a certain research foundation for demonstration learning in terms of application scenarios and research methods. Robot learning from demonstration can be divided into three parts: (1) How to demonstrate? (2) How to understand the demonstration of human beings. (3) How to learn from human demonstration.

Compared with traditional demonstration methods based on real environments, virtual reality [8] technology has many advantages. Nowadays, the application scenarios of virtual reality technology are becoming increasingly extensive, and the digital twin [9] technology in the industrial field constructs an increasingly complete digital copy of the real production environment, which can construct virtual scenes and establish task knowledge with minimal cost. In addition, the virtual reality system can record all the demonstration data of human experts. In terms of assembly tasks, how to achieve realistic physical assembly effects in a virtual environment is a difficult problem?

Although virtual assembly [10] has been a topic of widespread concern in academia for more than 20 years, however its practical application in industry is still limited. We believe this is due to the following reasons. First, most existing virtual assembly applications use unnatural user interfaces for control. Second, dynamic, physical assembly simulation cannot be realised. To this end, this paper designs and implements a virtual reality demonstration program for mechanical assembly. The application will solve the above problems by providing a natural user interface and a combination of dynamic assembly. In the dynamic simulation, physical components can be carried out. In order to create an assembly simulation that is both physically persuasive and convenient for hand tracking, we use the combination of real-time physical technology to achieve natural interaction between objects and kinematic constraints, thereby simplifying the assembly operation.

In summary, contributions of this paper include the following.

1. We construct a virtual reality demonstration system that uses virtual reality equipment for assembly activities demonstration.
2. We built a module for activity recognition and reasoning of human demonstrations.
3. The action sequence can be used as knowledge to transfer to different robots in different environments to perform the same task goal.

## 2. Related Works

### 2.1. Virtual Assembly

For many years, assembly training and virtual simulation based on virtual reality have been a topic of widespread concern in academia. Virtual assembly methods can be roughly divided into two types of commonly used methods: physical-based modelling (PBM) and constraint-based modelling (CBM). In the former method, physical interaction occurs between objects, and the connection between the parts is completed by physical contact between the parts. However, in CBM, the assembly is performed by introducing physical constraints, which reduces the degree of freedom between the objects to be manipulated, thereby restricting the relative position and rotation between the parts.

Physics-based modelling: Although physics-based modelling can provide high-precision simulation of assembly, the accuracy of physical simulation depends on the accuracy of the model used. Behandish et al. developed a general effective force model for virtual assembly [11]. Their method uses the artificial energy field around the virtual object to detect collisions between parts, and guides the parts to the required spatial configuration during the virtual assembly process. A major achievement of their method is the ability to unify the two phases of free movement and insertion into the body.

Constraint-based modelling: Although PBM focuses on providing realistic, physically-based interactions between parts, CBM sacrifices some physical precision to obtain other benefits, such as increased stability, better performance, accuracy, and simplicity. In the case of virtual assembly, CBM may be particularly useful, because the use of virtual constraints may be helpful to the user when physical constraints are missing. Wang et al. discussed the basis of constraint motion simulation and provided methods and algorithms for checking and applying constraints in assembly simulation [12]. In their research, they studied the analysis of the combination of axis and plane constraints and maintained the previously added constraints throughout the assembly process. They also proposed a way to guide users through visual display constraints during the assembly process. Murray et al. developed a virtual environment in the research on immersive assembly and maintenance simulation environment for constraint-based assembly and maintenance task simulation and analysis of large mechanical products [13].

In addition to PBM and CBM, hybrid modelling has also proposed a variety of hybrid methods that try to combine the advantages of the two methods. Tching et al. used the Virtuose tactile device to build an interactive simulation of CAD model assembly [14]. In addition to using mechanical joints to limit the relative movement between two parts, they also use virtual walls to guide objects to specific spatial configurations. They also formalised this concept as virtual constraint guidance (VCG) for insertion tasks. Although their approach is closer to CBM than PBM, they allow physical contact between virtual parts until the insertion task reaches a certain state. Seth et al. [15] also used a similar approach to combine PBM and CBM. They used the B-Rep solid model data in the CAD model data for collision detection between virtual parts and obtaining geometric constraints. Their application uses a custom physics engine for simulation. Although this method provides accurate simulation, we do not know of any available physics engine that will support B-Rep-based collision detection.

### 2.2. Activity Recognition

Although the research of using virtual reality technology in product organisation has been carried out for more than 20 years, the actual industrial application has not been widely used.

Compared with traditional demonstration methods based on real environments, virtual reality technology has many advantages. Nowadays, the application scenarios of virtual reality technology are becoming increasingly extensive, and the digital twin technology in the industrial field constructs an increasingly complete digital copy of the real environment. It is possible to construct virtual reality scenes and establish task knowledge with minimal cost. In addition, the virtual reality system can record all demonstration data of human experts, including direct motion data, operation event data, virtual sensor observation data and voice command data. These multi-source data provide strong support for the agent to learn operating skills and can be used for teaching robot. Different from the research work that uses real scenes for operation demonstrations, virtual reality systems are flexible, can easily change the environment and task scenes, and provide comprehensive and accurate motion data and access to underlying physical events. The latter means that virtual objects are Visibility, status, hierarchical relationships and force contact events between virtual objects can be read directly from the physics engine.

Activity recognition is the basic problem of the generation of agent skills, and its ultimate goal is to recognise new actions that it does not have and to have the ability to learn online. Aksoy et al. [16] proposed an action learning system based on the physical relationship between objects during operation. The system can extract the general definition of a specific action and a transformation matrix that describes the relationship between the actions. Summers-Stay et al. [17] used a simpler detection and segmentation method, but used a tree structure to describe the actions, which also captured the dependencies between the actions. The latest work in this field focuses on one-size and zero-sample learning techniques [18,19], which aims to minimise the number of training data sets and reduce the dependence on training data sets. The extreme case is the system ability to classify previous data and to recognise invisible movements without prior training. Cheng et al. proposed a framework that identifies new action categories based on human-readable semantic terms describing target activities [20]. However, not all operations can be easily described in text-based terms. Antol et al. developed a system that can learn from illustrations of required activities and can later apply the learning results to classify photographic images [21]. Even in the case of zero shots, many works still maintain a strong distinction between training and recognition, and may not be able to be learned instantly. Aoki et al. recently proposed an algorithm for unsupervised online learning of semantic information related to language terms and physical objects [20], in which robots can learn directly from humans through long-term interactions without prior training.

The work of Nuactiv [18] and later Cheng et al. [21] solved the problem of recognising actions related to the overall state of the human body. The work of the latter showed better recognition ability by considering the temporal relationship between semantic attributes used for classification. Aggarwal and Ryoo also considered complex time structures in order to use a layered approach to perform semantic analysis between human motion and object attributes to identify high-level activities [22]. Recently, decision trees have been used with powerful reasoning methods [23] and graphical models [24] to successfully learn functional object categories to infer human activities from demonstrations. The system of Dianov et al. [24] not only learns to recognise new activities, but also generates a high-level representation of the task space explored by the user, which can be used for robot planning.

Haidu and Beetz introduced a virtual reality-based skill learning environment for daily life operations in [25], which allows users to use Razer Hydra controllers to control virtual hands. From the many records of humans performing tasks in their environment, they are able to train (simulated) robotic agents to perform the same tasks. Ramirez-Amaro et al. also demonstrated advanced action recognition and learning in a virtual environment [26], which produces skill representations that can be transferred to physical robots. They used the SIGVerse [27] environment to capture the performance of participants and compared their results with similar experiments conducted using physics labs and camera-based tracking [23].

Ramirez-Amaro [28] provides a realistic, messy VR environment for experimental home tasks, plus a semantic extraction and reasoning system that can use real-time collected data and apply ontology-based reasoning to learn and classify activities. The system performs continuous segmentation of the user’s hand movements, and while classifying known movements, it learns new movements as needed. Then, the system constructs graphs of all relevant activities in the environment through observation and extracts the task space used by the observed users in the execution process. Compared with earlier work in physical and virtual spaces, the action recognition and learning system can maintain a high degree of accuracy of approximately 92 percent while dealing with more complex and realistic environments.

Michael Beetz [29] proposed a system that can collect and annotate daily activities performed by humans and understandable by robots from a virtual environment. Using off-the-shelf virtual reality equipment with full-body functions and eye-tracking functions, it is possible to draw human movement diagrams in a simulated world. All interactions in the virtual world are physically simulated, so movement and its effects are closely related to the real world. During the execution of the activity, a sub-symbol data recorder is recording the environment and people’s sight frame by frame, thereby realising the reproduction and playback of offline scenes. Combined with the physics engine, the online monitor (symbolic data recorder) is parsing (using various grammars) and recording events, actions and their effects in the simulated world.

Constantin Uhde [30] introduces a novel learning method for extracting tool dependencies by following the scientific cycle of observation, generating causal hypotheses and testing through experiments. They use a virtual reality data set containing observations from human activities to generate hypotheses about causality between actions. It detects action pairs that appear at the same time in time, and verifies whether one action helps to perform another action through mental simulation in a virtual reality environment that represents the mental model of the system. The proposed method can extract all current tool action dependencies, while significantly reducing the search space for mental simulation, thereby reducing the calculation time by 6 times.

## 3. Methodology

### 3.1. System Overview

**Figure 1:**
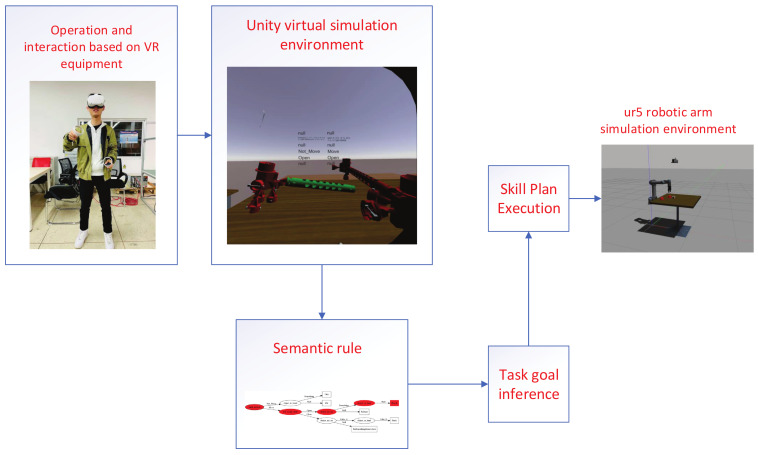
System overview.

In the research of this article, we propose a framework for inferring the semantics of activities based on virtual reality demonstrations. This framework includes three main modules: (1) Assembly task demonstration based on virtual reality helmet and unity physics engine. (2) Semantic activity decomposition of the demonstration task. (3) Semantic activity sequences of perception are performed by the robotic arm. Figure 1 describes our framework and the relationship between these frameworks.

The first module is the virtual reality task demonstration module. We use the game physics engine Unity to build a simulation environment for the assembly of workpieces. It is difficult to simulate a real assembly task in a virtual environment because the objects in the virtual environment have no real physical properties, such as friction. In order to imitate the real assembly effect as much as possible, we designed the link method of the part assembly.

The second module is responsible for the analysis and processing of sensor data in the virtual environment. The motion state of the hand is segmented from the motion data. In the virtual assembly environment, we can directly obtain the position information of the hands and objects in the environment, and the opening and closing states of the hands can be read from the buttons of the virtual reality device controller. Based on these sensor data, we set four attribute values for inferring the semantics of single hand activities. We have designed three additional attributes to define the assembly activities of double hands. For example, we define the hand-motion attribute to determine whether the hand is moving, including two attribute values: move and not-move.

The third module is a simulated UR5 robotic arm grasping system. The inferred activity sequence can be tested on the simulated system.

### 3.2. Virtual Assembly Environment

**Figure 2:**
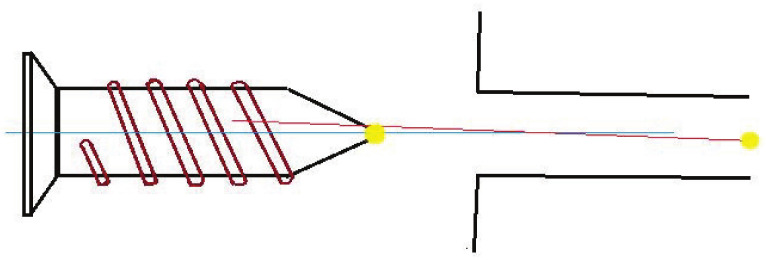
Pin in hole assembly activity.

We use the game physics engine Unity to build the virtual environment of industrial assembly. Take truss assembly as the task scene of the experiment. It is very difficult to simulate the screwing link of two objects in a virtual environment. To this end, this paper builds a set of software and hardware systems for interactive virtual assembly, as shown in Figure 2, including a set of VR equipment for human operators to interact with the virtual environment, and a digital twin environment for industry assembly task scenarios. The VR device used in this article is Oculus Quest2, which includes a VR helmet and a pair of control handles. After the operator is running the system, the operator wearing the VR helmet can see the assembly process table in the virtual environment from the first-person perspective, and control handle is responsible for manipulating the virtual hand in the virtual scene to perform assembly tasks.

In this paper, interactive assembly simulation is performed in an immersive virtual environment. Fidelity is the focus of the virtual assembly environment. Therefore, 3DMax is used to build a virtual assembly environment model to ensure the realistic effects of the virtual assembly environment. After completing the modelling in 3DMax according to the dimensions of the real parts of the assembly and the connectors, etc., export the file in Fbx format to Unity3D. When the model required for assembly is imported into Unity3D, taking into account that in the process of assembling each sub-assembly into a total assembly, all parts on the sub-assembly need to move or rotate together, so it is necessary to perform structure processing for the original model, strictly define the parent–child relationship between components.

As it is more difficult to perform fine assembly operations in the virtual scene, we have defined a connector mechanism to help complete the connection of the parts when completing the assembly connection of the parts. All the parts available for assembly have dumbbell-shaped geometry. There is a square at each end of the geometry. We define the dumbbell-shaped geometry as a connector and the cube as a connector-cube. One of the connector blocks is used for collision detection, called (connector-cube-collide), after this referred to as CCC, and the other connector block is used for assembly positioning, called (connector-cube-orientate), after this referred to as CCO. As a part may need to be assembled and connected with other parts of different quantities and types at the same time, the number and types of connectors on different parts are not the same.

When performing assembly operations in a virtual environment, if the CCCs of a pair of parts collide are matched, and the following conditions are met, the connector-mechanism is executed to complete the connection of the parts and form a combination:

Condition 1: The Euler distance between the geometric centres of two matching connectors CCC A and CCC B is less than the threshold.

(1) $$d_{L2}\left({\left(x,y,z\right)}_{A},{\left(x,y,z\right)}_{B}\right)<\varepsilon_{d}$$

where ${\left(x,y,z\right)}_{A}$ is the three-dimensional coordinates of the centre of connector A, ${\left(x,y,z\right)}_{B}$ is the three-dimensional coordinates of the centre of connector B, $d_{L2}\left({\left(\right)}_{A},{\left(\right)}_{B}\right)$ is the Euler distance between the centre of the connector A and the connector B and $\varepsilon_{d}$ is the preset Euler distance threshold.

Condition 2: The cosine distance of the unit axial vector between the geometric centres of two matching connectors A and connector B is less than the threshold.

(2) $$\begin{array}{c} d_{forward}+d_{up}<\varepsilon_{\cos}\\ d_{forward}=1-\frac{V_{A\_forward}\cdot V_{B\_forward}}{\left|V_{A\_forward}\right|\left|V_{B\_forward}\right|}\\ =1-\frac{x_{A\_forward}*x_{B\_forward}+y_{A\_forward}*y_{B\_forward}+z_{A\_forward}*z_{B\_forward}}{\sqrt{x_{A\_forward}^{2}+y_{A\_forward}^{2}+z_{A\_forward}^{2}}*\sqrt{x_{B\_forward}^{2}+y_{B\_forward}^{2}+z_{B\_forward}^{2}}}\\ d_{up}=1-\frac{V_{A\_up}\cdot V_{B\_up}}{\left|V_{A\_up}\right|\left|V_{B\_up}\right|}=1-\frac{x_{A\_up}*x_{B\_up}+y_{A\_up}*y_{B\_up}+z_{A\_up}*z_{B\_up}}{\sqrt{x_{A\_up}^{2}+y_{A\_up}^{2}+z_{A\_up}^{2}}*\sqrt{x_{B\_up}^{2}+y_{B\_up}^{2}+z_{B\_up}^{2}}} \end{array}$$

where $d_{forward}$ is the cosine distance between the *Z*-axis unit vectors of the two vectors’ own coordinate system, $V_{A\_forward}$ is the unit vector of the *Z*-axis of the connector A’s own coordinate system, $V_{B\_forward}$ is the unit vector of the *Z*-axis of the connector B’s own coordinate system, $\left(x_{A\_forward},y_{A\_forward},z_{A\_forward}\right)$ is the specific vector coordinates of $V_{A\_forward}$ and $\left(x_{B\_forward},y_{B\_forward},z_{B\_forward}\right)$ is the specific vector coordinates of $V_{B\_forward}$; and $d_{up}$ is the cosine distance between the *Y*-axis unit vectors of the two vectors’ own coordinate system, $V_{A\_up}$ is the unit vector of the *Y*-axis of the connector A’s own coordinate system, $V_{B\_up}$ is the unit vector of the *Y*-axis of the connector B’s own coordinate system, $\left(x_{A\_up},y_{A\_up},z_{A\_up}\right)$ is the specific vector coordinates of $V_{A\_up}$ and $\left(x_{B\_up},y_{B\_up},z_{B\_up}\right)$ is the specific vector coordinates of $V_{B\_up}$. $\varepsilon_{\cos}$ is the preset cosine distance threshold.

Take the assembly of Phillips screws and screw holes as an example. The two circles marked in yellow in the figure represent the connectors of Phillips screws and screw holes, respectively. When the difference between the CCC centroid distances of the two connectors is less than the set threshold and the centre axis (the blue and red lines in the figure represent the *Z* axis of its own coordinate system, the *Y* axis is not drawn in the same way), the relative difference between the directions is not large ( When the cosine distance is less than the set threshold), set the status of the Phillips screw and screw hole to “connectable”. At this time, the connector mechanism can be activated, and the CCO starts its assembly positioning function and enters the automatic assembly stage. The screw moves along the *Z* axis of its own coordinate system until the CCO of the screw and the CCO of the screw hole are completely overlapped, and the assembly of the screw and the screw hole is completed.

### 3.3. Define and Extract Virtual Sensor Information

How to turn the tasks demonstrated by humans into information that robots can understand is a complex problem. In the virtual demonstration environment, the motion data of the demonstrator can be directly obtained. We need to process these motion data to obtain meaningful activity semantic information. We refer to the method proposed by Ramirez-Amaro [26] and make improvements and enhancements on this basis. Ramirez-Amaro’s method is to extract spatio-temporal features from video data and then perform staged action recognition. In addition, Ramirez-Amaro’s method is aimed at human activities in daily life, and it is difficult to analyse complex industrial assembly operations.

Compared with extracting spatio-temporal features from the video, in the virtual assembly environment, we can directly obtain more intuitive and accurate motion data, which has great advantages. For example, we can read the opening and closing status of the virtual hand through the oculus controller, which is difficult to obtain in video data. For the three-dimensional demonstration activities in the virtual environment, we can obtain the continuous position change information of the hand. We define the hand-motion attribute variable to have two attribute values: move and notmove. Through the Oculus Quest2 controller buttons, we can directly obtain the open and close status of the hand. In addition, we also need to obtain information about the interaction between the hand and the objects in the virtual environment. The attributes that can be easily identified from observation are ObjectActedOn (oa) and ObjectInHand (oh). Define the ObjectInHand property as the object grabbed by the virtual hand, and ObjectActedOn as the object approached by the virtual hand. Table 1 lists the four attributes and the corresponding attribute values.

### 3.4. Infer the Activity Semantics of the Demonstrator

In this article, the semantics of human behaviour refers to finding a meaningful relationship between human motion and motion attributes in order to understand the activities performed by humans. For industrial assembly activities, we define the basic activities of industrial assembly, including reaching, taking, moving, releasing and other activities. We want to do continuous motion segmentation for the demonstration of the virtual environment. Under the refresh rate of unity, the current activity of the demonstrator can be analysed every frame. Different activities will have different durations. With a high refresh rate, very short-lived activities can also be sensed, and no activity loss will occur.

Motion segmentation is performed separately for each hand and follows the description of Ramirez-Amaro [26] et al. The attributes in Table 2 describe the activities defined by hand segmentation. The opening and closing of the hand can be read directly from the state of the oculus controller.

In order to calculate the value of these attributes, we have made some restrictions. For example, the hand cannot act on the object currently held by the hand.

Human assembly activities generally target two objects. To this end, on the basis of single-handed activity recognition, we further define two-handed collaborative activities, as shown in Table 3. First, define three new activity attributes: object distance is used to describe the distance between the two objects to be assembled, and Connect-flag is used to indicate whether the two objects to be assembled meet the conditions of assembly. Collide-flag is used to indicate that two objects are colliding but do not meet the assembly conditions.

Under the condition that the ObjectInHand of both hands is not empty, we can define two-handed collaboration activities, as shown in the following Table 4.

### 3.5. Decision Tree

The decision tree classifier is used to learn the mapping between human motion and human behaviour based on motion information. In order to train the learned decision tree, we collect sample data $\left(s,c\left(s\right)\right)$ for training, such as

(3) $$\left(\left[\begin{array}{cccc} NotMove & Something & Null & Close \end{array}\right],Take\right)$$

where *s* is a vector that stores the attribute value of the current activity, and $c\left(s\right)$ is the activity corresponding to the current movement state. In order to learn the objective function from a set of training examples *s*, we use the C4.5 algorithm to calculate the decision tree.

**Figure 3:**
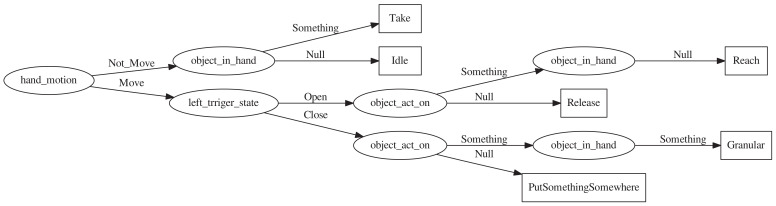
Decision tree.

**Figure 4:**
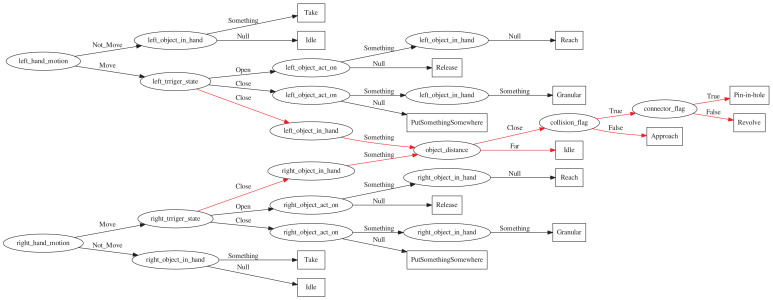
Extended decision tree, the red parts are the two-hand assembly activities.

The decision tree generated in this study is shown in Figure 3. It consists of two steps: the first step is to generate a tree, which can determine the basic human hand activities in a general form (that is, reach, take, putsomethingsomewhere, release and idle). The second method extends the tree obtained by the tree to identify more complex two-hand assembly activities. We refer to these types of activities as granular activities, such as approach, revolve and pin-in-hole, as shown in Figure 4.

### 3.6. Transfer the Task to the Robot and Execute

Obtaining the semantics of the activities through the semantic activity inference module, we also need to perform tasks on the robotic arm. We use Gazebo to build a ur5 robotic arm simulation system to execute our algorithm. The control system must be able to perform inferred activities quickly and be free from interference from noise in the environment. Therefore, transferring the model we obtained to the robot is considered to be another important contribution of this paper.

The last module integrates data input from multiple sources. These data include: virtual assembly demonstration, a robotic arm simulation platform. Specifically, we used gazebo to build a simulation environment for the ur5 collaborative robotic arm, and integrated the perception and reasoning module into the motion planning of the robotic arm. In order to achieve this goal, we need to solve several problems. The first is that the perception and reasoning module needs to work online. Second, the perception and reasoning module must be as fast and accurate as possible. This requires that the communication between the perception module and the reasoning module must be real-time, because these modules work with the motion planning module of the robotic arm.

**Figure 5:**
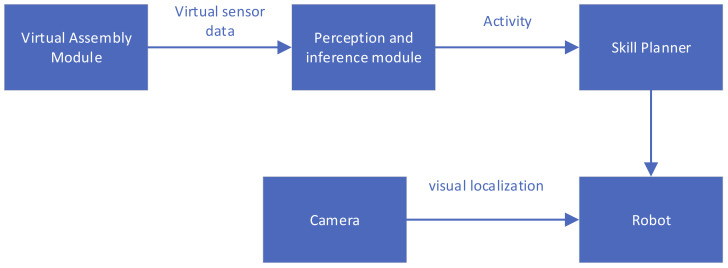
Transfer the demonstration task to the robotic arm for execution.

Figure 5 shows the different components of the robotic arm execution system. The camera module provides the function of visual localisation, recognises objects in the scene and determines the location of the objects. The perception and reasoning module parses the virtual demonstration into activity instructions in real-time, and the skill planning module stores the motion primitives that should be executed to achieve tasks similar to the observed tasks. For example, after the robot infers human behaviour (g), it visits the skill planning module, where a series of primitive sequences are stored. This list of primitives defines specific tasks. The output of this module includes execution primitives (pn) from the library in the retrieved plan, where n is the number of executed primitives. For example, if the inferred activity has arrived, the skill plan will order primitives: p1 = find the desired object, p2 = approach the object and so on. The control loop performs this control until the required task is successfully completed. Then, load and execute the next primitive in the same form. This process continues until the last primitive is generated. Examples of controls used in this article include: joint space controls using inverse kinematics and visual positioning controls.

Two main modules are implemented in the thread loop: online activity inference and robot execution module.

The online activity inference model divides and interprets the visual demonstration data from the virtual environment. The box highlighted in red in the figure represents the new function we have implemented in the robot, which is an explanation of the human observation. These abilities will trigger (online) the motion primitives that the robot needs to execute in order to achieve goals similar to the observed goals.

The robot execution module obtains visual information from the robot’s camera to detect objects in its work area. This information is mapped to the joint space and used as feedback for the control loop. It is important to note that the modular architecture of the framework allows us to replace any module with more complex behaviours obtained. For example, the vision module can be replaced with a more advanced detection system, or the control method can be replaced with a more powerful Adaptive control law, for example [31]. The function converts the observed human behaviour into robot actions, as shown below.

(1) We define a plan execution library, which has a given goal (g), and primitives (p) can be selected from the library that must be executed by the robot. For example, if the inferred goal has been reached, the execution plan will include the following steps: (1) find the goal (o1), (2) determine the location of o1 and (3) move the hand to o1.

(2) From the execution plan, we obtain the n primitives (p(n)) that the robot needs to execute. These primitives are retrieved from the primitive database. According to this example, the first primitive p(1) will trigger the camera’s target detection algorithm. Next, p(2) will find the object, and the robot controller will retrieve the fixed point. Finally, p(3) will allow the Cartesian interface of the robot to control the arm in the joint space (q).

(3) Perform these steps until the last step of the execution plan is completed.

## 4. Results and Discussion

### 4.1. Semantic Representation of Results

Typical industrial assembly scene tasks can be broken down into some basic activities, such as approaching, grabbing, moving and releasing. These activities can form an activity chain diagram of tasks.

**Figure 6:**
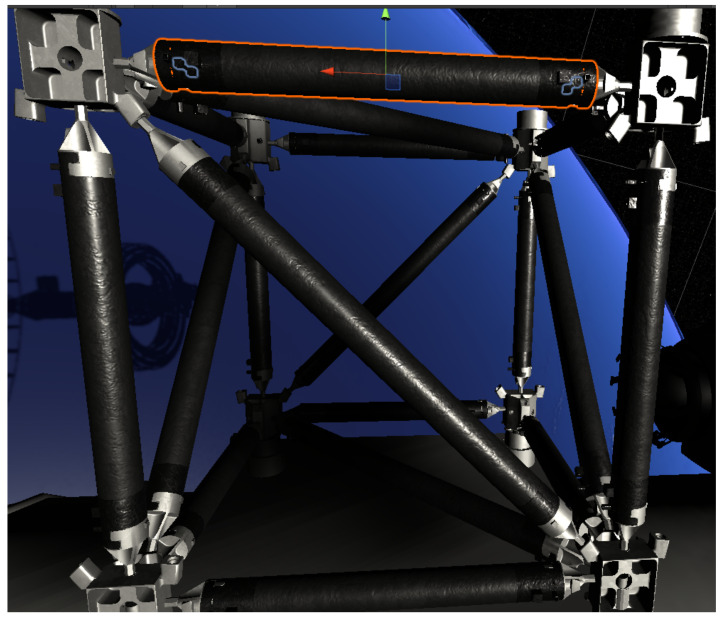
Space trusses scene.

This article takes the assembly of space trusses as an application scenario, as shown in Figure 6. In outer space, robots are required to perform maintenance or assembly tasks on equipment. The assembly of the truss is mainly the assembly of rods and nodes.

**Figure 7:**
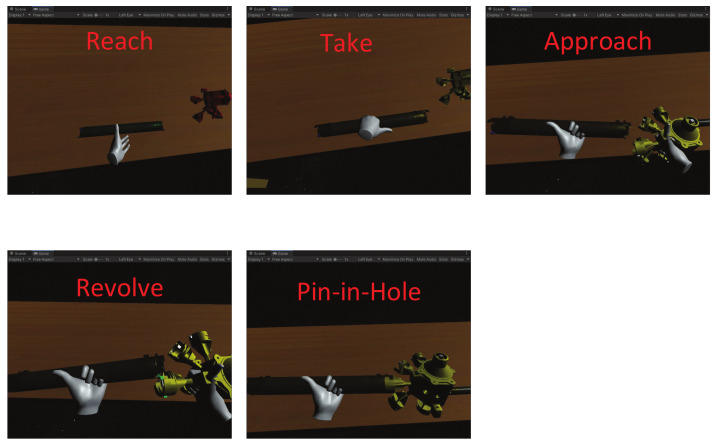
Activity inference for the demonstration scenario of the truss assembly task.

We invite eight experimenters without VR interaction experience. The participants in the experiment all got the task command, “Please use the virtual hand to complete the assembly of the truss.” For the experimenter, the only difficulty in the assembly process is to adjust the two parts to be assembled to the appropriate position and direction. When the correct position and orientation are met, let go of the virtual hand, and the two parts to be assembled can be automatically assembled together. In order to complete the assembly task, the demonstration activities of the demonstrator are deduced into the following activities, as shown in Figure 7.

By playing back the recorded VR data and manually performing motion segmentation and activity semantic classification. By simply evaluating each time step in the log, the basic facts can be evaluated. Any point in time when the detected value and the true value are inconsistent is regarded as an error.

In order to evaluate the accuracy of activity recognition, we collect first-person demonstration images of testers divide them into different segments according to commands during the tester’s demonstration process. Ground truths for the sessions were constructed by playing back the recorded VR data and manually performing motion segmentation and activity classification. Finally, by comparing with these ground-truth data, it is concluded that the average correct rate based on the activity inference algorithm is 95%. The detailed accuracy of each activity is shown in the confusion matrix shown in Table 5, where the main diagonal representation of the form indicates that the judgement of human activity in most clips is correct.

### 4.2. Transfer the Target to the Robot and Execute

Several experiments were carried out in the gazebo simulation environment of the ur5 robotic arm. Our system comprises two subsystems: (1) activity observation and interpretation of human activities and (2) robotic arms to perform activities. These subsystems have been implemented in the UR5 control system.

**Figure 8:**
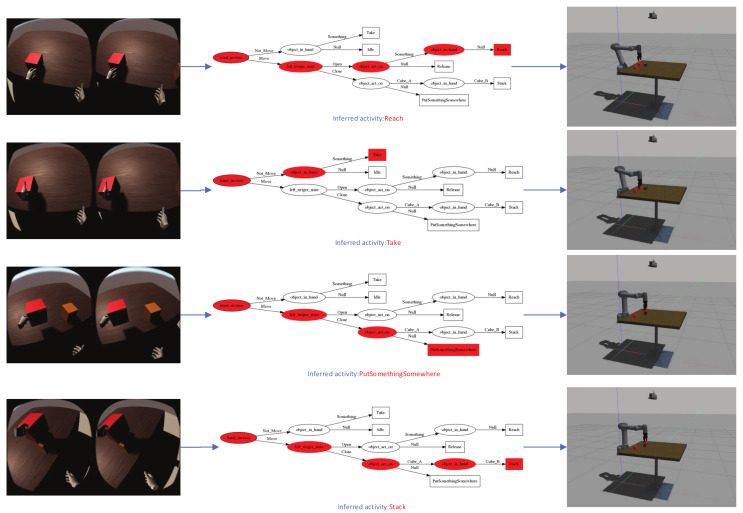
Activity performed by a human and that inferred and executed by the UR5 robot arm.

Figure 8 shows some examples of the system results we implemented on UR5. In this figure, the first row shows that the perception system will grab and place the virtual presentation to retrieve the recognised high-level motion and object properties. Take the stacking of wood blocks as an example. In the first row of Figure 8, motion = move and objectActedOn(oa) = CubaA. Then, immediately infer the human goal. This shows that as shown in the first line of Figure 8, a branch of the tree obtained from Figure 3 was executed. In other words, human activity is inferred by the system, in this case activity = Reach is sent to the robot to be executed. Then, execute the doReach() execution plan, as shown in the first line of Figure 8.

Similar to the previous example, Figure 8 depicts more activities inferred and performed by UR5 in real-time. For example, the second row of Figure 8 shows the activity of grabbing an object, and the third row shows the activity of placing something somewhere. The last line shows the stacking activity; note that this represents a new activity of the system in this case. This means that the activity is learned on demand. Please note that all implemented applications and modules in our system are running during execution.

## 5. Conclusions

For robots, understanding human demonstration activities is a complex issue. By building a virtual reality demonstration system for simulating industrial assembly, we can obtain the demonstration data of the demonstrator in a natural and convenient way and use the obtained data to understand the activity of the demonstrator. The activity inference module decomposes the demonstration data of the demonstrator into task graphs required to complete the task. The task graph can be used to guide the real robot to reproduce the task, and can be transmitted in different environments and robots.

The current virtual reality demonstration system is still relatively simple, just simulating the jack task demonstration and activity inference. We hope to further expand our work in the following aspects. One is to increase the fidelity of the virtual environment, and the physical effects of the scene should also be considered. How to reflect physical parameters such as friction and gravity in the virtual environment is a difficult problem. The second is to add robots to the current virtual demonstration system to realise the virtual demonstration of human and robot cooperation to complete tasks, which can provide great help to human–robot safety cooperation.

## Figures and Tables

**Table 1:** One-handed activity attributes.

Attributes	Moving	ObjectInHand	ObjectActOn	HandState
	${\dot{x}}_{hand}>c$	$\left|x_{obj}-x_{hand}\right|=0$	$\left|x_{obj}-x_{hand}\right|\to 0$	
Value	Move	ObjectName	ObjectName	Open
	NotMove	Null	Null	Close

**Table 2:** Primitive activity definitions.

Activity	Moving	ObjectInHand	ObjectActOn	HandState
Idle	NotMove	Null	Null	Open
Reach	Move	Null	Something	Open
Take	NotMove	Something	Null	Close
Release	Move	Null	Null	Open
PutSomethingSomewhere	Move	Something	Null	Close
Granular	Move	Something	Something	Close

**Table 3:** Double hands activity attributes.

Attributes	ObjectDistance	Collide-Flag	Connect-Flag
	$\left|x_{obj1}-x_{obj2}\right|<\alpha$	Collision of two objects	Meet the assembly conditions
	far	True	True
	close	False	False

**Table 4:** Two-handed collaboration activities.

Activity	Moving	ObjectDistance	Collide-flag	Connect-flag
Idle	NotMove	far	False	False
Approach	Move	close	False	False
Revolve	Move	close	True	False
Pin-in-Hole	Move	close	True	True

**Table 5:** Confusion matrix (expressed in %) obtained from the space truss assembly, and ground-truth data, where a = Reach, b = Grasp, c = Release, d = Idle, e = Approach, f = Revolve, and g = Pin-in-hole.

Actual Activities			Inference Activities				
	a	b	c	d	e	f	g
**a**	95.5	4.1	0	0.3	0	0	0
**b**	20.5	94.3	2.3	3.4	0	0	0
**c**	0	0	98.6	1.4	0	0	0
**d**	0	0	8.8	91.2	0	0	0
**e**	0	0	0	1.2	98.7	0	0
**f**	0	0	0	0	0	96.4	3.5
**g**	0	0	0	0	0	3.3	96.7

## Data Availability

Not applicable.

## References

[1] Ravichandar H., Polydoros A.S., Chernova S., Billard A. (2020). Recent Advances in Robot Learning from Demonstration. Annu. Rev. Control. Robot. Auton. Syst..

[2] Siciliano B., Khatib O. (2016). Learning from Humans.

[3] Ambhore S. A Comprehensive Study on Robot Learning from Demonstration. Proceedings of the 2020 2nd International Conference on Innovative Mechanisms for Industry Applications (ICIMIA).

[4] Finn C., Yu T., Zhang T., Abbeel P., Levine S. One-shot visual imitation learning via meta-learning. Proceedings of the Conference on Robot Learning.

[5] Sheh R.K.M. “Why Did You Do That?” Explainable Intelligent Robots. Proceedings of the Workshops at the Thirty-First AAAI Conference on Artificial Intelligence.

[6] Kim B., Pineau J. (2016). Socially Adaptive Path Planning in Human Environments Using Inverse Reinforcement Learning. Int. J. Soc. Robot..

[7] Goodfellow I.J., Pouget-Abadie J., Mirza M., Xu B., Warde-Farley D., Ozair S., Courville A., Bengio Y. (2014). Generative Adversarial Networks. Adv. Neural Inf. Process. Syst..

[8] Aleotti J., Caselli S. Grasp recognition in virtual reality for robot pregrasp planning by demonstration. Proceedings of the 2006 IEEE International Conference on Robotics and Automation.

[9] Lv Q., Zhang R., Sun X., Lu Y., Bao J. (2021). A Digital Twin-Driven Human-Robot Collaborative Assembly Approach in the Wake of COVID-19. J. Manuf. Syst..

[10] Gonzalez-Badillo G., Medellin-Castillo H., Lim T., Ritchie J., Garbaya S. (2014). The development of a physics and constraint-based haptic virtual assembly system. Assem. Autom..

[11] Behandish M., Ilieş H.T. (2015). Peg-in-hole revisited: A generic force model for haptic assembly. J. Comput. Inf. Sci. Eng..

[12] Wang Y., Jayaram U., Jayaram S., Imtiyaz S. (2003). Methods and algorithms for constraint-based virtual assembly. Virtual Real..

[13] Murray N., Fernando T. An immersive assembly and maintenance simulation environment. Proceedings of the Eighth IEEE International Symposium on Distributed Simulation and Real-Time Applications.

[14] Tching L., Dumont G., Perret J. (2010). Interactive simulation of CAD models assemblies using virtual constraint guidance. Int. J. Interact. Des. Manuf. (IJIDeM).

[15] Seth A., Vance J.M., Oliver J.H. (2010). Combining dynamic modeling with geometric constraint management to support low clearance virtual manual assembly. J. Mech. Des..

[16] Aksoy E.E., Abramov A., Dörr J., Ning K., Dellen B., Wörgötter F. (2011). Learning the semantics of object–action relations by observation. Int. J. Robot. Res..

[17] Summers-Stay D., Teo C.L., Yang Y., Fermüller C., Aloimonos Y. Using a minimal action grammar for activity understanding in the real world. Proceedings of the 2012 IEEE/RSJ International Conference on Intelligent Robots and Systems.

[18] Cheng H.T., Sun F.T., Griss M., Davis P., Li J., You D. Nuactiv: Recognizing unseen new activities using semantic attribute-based learning. Proceedings of the 11th Annual International Conference on Mobile Systems, Applications, and Services.

[19] Antol S., Zitnick C.L., Parikh D. (2014). Zero-shot learning via visual abstraction. European Conference on Computer Vision.

[20] Aoki T., Nishihara J., Nakamura T., Nagai T. Online joint learning of object concepts and language model using multimodal hierarchical Dirichlet process. Proceedings of the 2016 IEEE/RSJ International Conference on Intelligent Robots and Systems (IROS).

[21] Cheng H.T., Griss M., Davis P., Li J., You D. Towards zero-shot learning for human activity recognition using semantic attribute sequence model. Proceedings of the 2013 ACM International Joint Conference on Pervasive and Ubiquitous Computing.

[22] Aggarwal J.K., Ryoo M.S. (2011). Human activity analysis: A review. ACM Comput. Surv. (CSUR).

[23] Ramirez-Amaro K., Beetz M., Cheng G. (2017). Transferring skills to humanoid robots by extracting semantic representations from observations of human activities. Artif. Intell..

[24] Dianov I., Ramirez-Amaro K., Lanillos P., Dean-Leon E., Bergner F., Cheng G. Extracting general task structures to accelerate the learning of new tasks. Proceedings of the 2016 IEEE-RAS 16th International Conference on Humanoid Robots (Humanoids).

[25] Haidu A., Beetz M. Action recognition and interpretation from virtual demonstrations. Proceedings of the 2016 IEEE/RSJ International Conference on Intelligent Robots and Systems (IROS).

[26] Ramirez-Amaro K., Inamura T., Dean-León E., Beetz M., Cheng G. Bootstrapping humanoid robot skills by extracting semantic representations of human-like activities from virtual reality. Proceedings of the 2014 IEEE-RAS International Conference on Humanoid Robots.

[27] Inamura T., Shibata T., Sena H., Hashimoto T., Kawai N., Miyashita T., Sakurai Y., Shimizu M., Otake M., Hosoda K. Simulator platform that enables social interaction simulation—SIGVerse: SocioIntelliGenesis simulator. In Proceedings of the 2010 IEEE/SICE International Symposium on System Integration.

[28] Bates T., Ramirez-Amaro K., Inamura T., Cheng G. On-line simultaneous learning and recognition of everyday activities from virtual reality performances. Proceedings of the 2017 IEEE/RSJ International Conference on Intelligent Robots and Systems (IROS).

[29] Kazhoyan G., Hawkin A., Koralewski S., Haidu A., Beetz M. Learning Motion Parameterizations of Mobile Pick and Place Actions from Observing Humans in Virtual Environments. Proceedings of the 2020 IEEE/RSJ International Conference on Intelligent Robots and Systems (IROS).

[30] Uhde C., Berberich N., Ramirez-Amaro K., Cheng G. The Robot as Scientist: Using Mental Simulation to Test Causal Hypotheses Extracted from Human Activities in Virtual Reality. Proceedings of the 2020 IEEE/RSJ International Conference on Intelligent Robots and Systems (IROS).

[31] James S., Johns E. (2016). 3D Simulation for Robot Arm Control with Deep Q-Learning. arXiv.

